# Adult dietary patterns and their association with iodine nutrition levels and thyroid function: a cross-sectional study

**DOI:** 10.1017/S1368980024002404

**Published:** 2024-11-27

**Authors:** Gulinaizeer Abuduwaili, Jia Huang, Yan Ma, Hongguang Sun

**Affiliations:** 1 School of Public Health, Xinjiang Medical University, Urumqi 830054, People’s Republic of China; 2 Institute for the Control of Pathogenic Organisms, Xinjiang Uyghur Autonomous Region Centre for Disease Control and Prevention, Urumqi 830002, People’s Republic of China

**Keywords:** Dietary patterns, Urinary iodine, Thyroid function, Iodine deficiency, Adult

## Abstract

**Objective:**

To understand the dietary patterns of adults and explore their association with iodine nutritional levels and thyroid function in adults.

**Design:**

We randomly collected 5 ml of adult urine samples and measured urinary iodine concentration (UIC) by cerium arsenate-catalysed spectrophotometry. A serum sample of 5 ml was collected for the determination of free triiodothyronine, free thyroxine and thyrotropin, and diet-related information was collected through a FFQ. Dietary patterns were extracted by principal component analysis, and the relationship between dietary patterns and iodine nutrition levels and thyroid function was explored.

**Settings:**

A cross-sectional study involving adults in Xinjiang, China, was conducted.

**Participants:**

A total of 435 adults were enrolled in the study.

**Results:**

The overall median urinary iodine of the 435 respondents was 219·73 μg/l. The dietary patterns were PCA1 (staple food pattern), PCA2 (fruit, vegetable and meat pattern), PCA3 (fish, shrimp and legume pattern) and PCA4 (dairy-based protein pattern). The correlation analyses showed that PCA1 and PCA3 were positively correlated with the UIC. The results of the multivariable analysis showed that PCA1, Q1, Q2 and Q3 were associated with an increased risk of iodine deficiency compared with Q4 ((OR): 260·41 (95 % CI: 20·16, 663·70)), 59·89 (5·64, 335·81), and 2·01 (0·15, 26·16), respectively). In PCA2, Q3 was associated with an increased risk of iodine deficiency compared with Q4 (OR: 0·16 (0·05, 0·53)). In PCA3, Q3 was associated with an increased risk of iodine deficiency compared with Q4 (OR: 0·23 (0·06, 0·90)). In PCA4, Q1 was associated with an increased risk of iodine deficiency compared with Q4 (OR: 31·30 (4·88, 200·64)).

**Conclusion:**

This study demonstrated that of the four dietary patterns, the least dependent staple food pattern (Q1) had a higher risk of iodine deficiency compared with the most dependent staple food pattern (Q4). However, the current evidence on the effect of dietary patterns on thyroid function needs to be validated by further longitudinal studies that include long-term follow-up, larger sample sizes and repeated measures.

Dietary intake is one of the most important factors affecting health, but it varies among individuals^(1)^. The development of society and the elevation of economic standards have led to a growing interest in understanding the complexity of dietary intake and its interactions with health outcomes, making it necessary to study the impact of different dietary patterns, as well as individual nutrients, on health. Dietary patterns consider the complex interrelationships between different foods or nutrients as a whole, reflecting the actual dietary habits of individuals and providing information on the association of nutrients with disease, if any^(2)^. In addition, dietary patterns are more consistent over time and have a greater impact on health outcomes than individual nutrients^(3)^.

Iodine, an essential trace element, is a crucial raw material for the synthesis of thyroid hormones, and an appropriate amount of iodine intake plays an important role in the growth and development of the human body and the maintenance of the normal function of the thyroid gland^(4)^. Previous studies have confirmed that there is a ‘U-shaped’ relationship between iodine intake and thyroid disease and that both iodine excess and iodine deficiency can cause abnormalities or diseases of the thyroid gland^(5)^. In regions with different degrees of iodine deficiency, individuals show a spectrum of mild to severe iodine deficiency, with corresponding clinical manifestations varying in different developmental stages^(6)^. Numerous studies have shown that iodine deficiency in children can affect their intellectual and physical development, causing motor, visual and hearing impairments, highlighted by goitre^(7)^. Iodine deficiency in pregnant women affects not only their own thyroid function but also the neurodevelopment of their offspring and can even lead to poor pregnancy outcomes^(8,9)^. Because children and pregnant women are especially susceptible to iodine deficiency disorders, numerous related studies in China have focused on these vulnerable populations, while little research has been conducted on iodine nutrition and thyroid function in adults. Iodine deficiency poses a risk to adults as well, who make up the vast majority of the population.

The global strategy to improve iodine deficiency through universal salt iodisation policy and the food iodine fortification initiative has achieved remarkable results. Since the implementation of universal salt iodisation in 1994, iodine nutrition in the Chinese population has improved significantly^(10)^. Xinjiang province, located in northwestern China, is classified as being iodine deficient in the external environment due to >90 % of its water sources having iodine levels below 10 μg/l^(11)^. In recent years, the living conditions of Xinjiang residents have improved, living standards have been elevated and health awareness has gradually increased; however, due to differences in geographic location, iodine supplementation measures and dietary habits between northern and southern Xinjiang, the distribution of iodine nutrition levels is uneven between their residents, with some residents still suffering from iodine deficiency. Therefore, the present study investigated the dietary patterns of adults in Xinjiang and explored the association of dietary patterns with their iodine nutrition levels and thyroid function.

## Materials and methods

### Participants and study design

**Figure 1.**
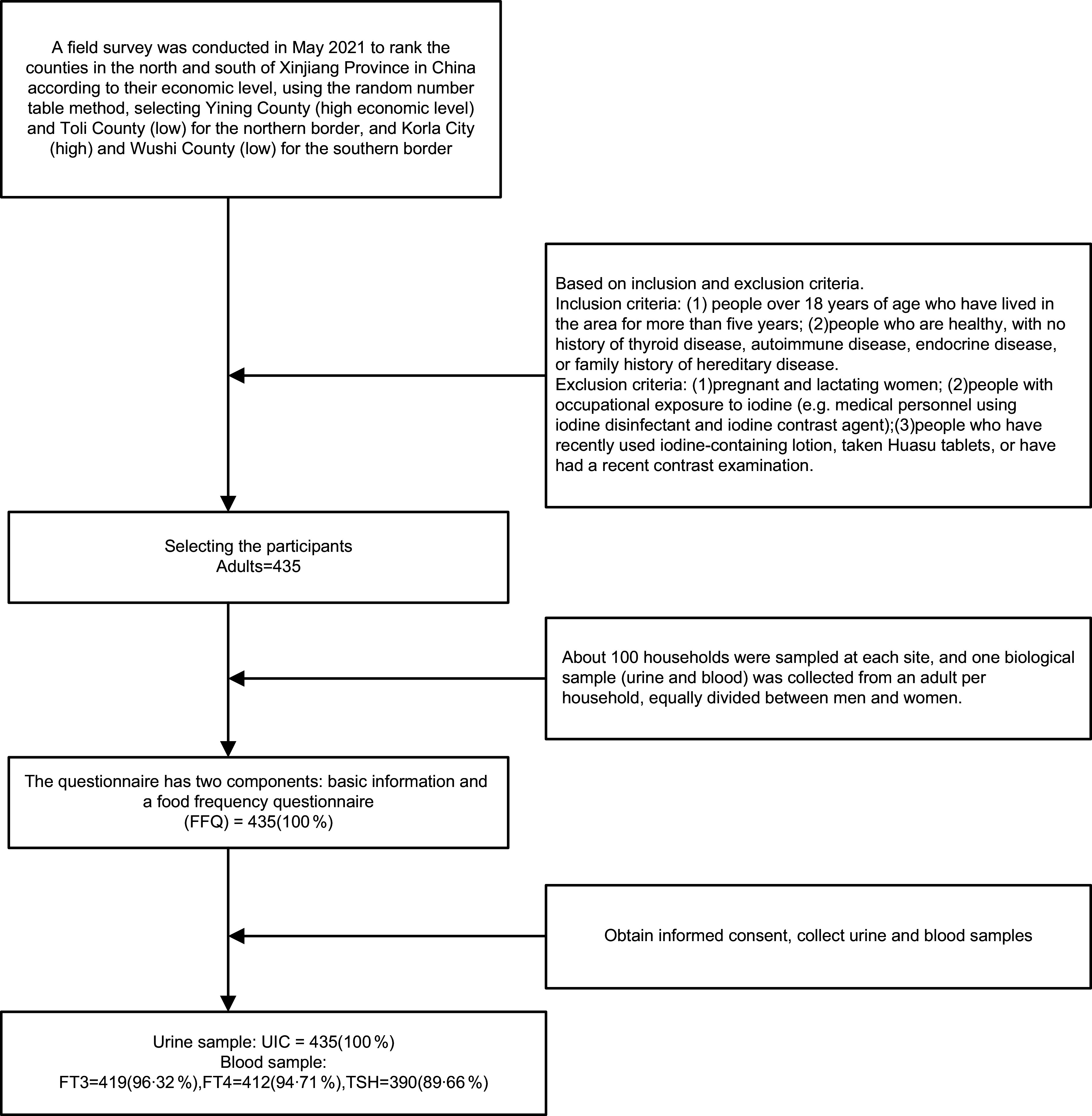
Participant flow chart. This figure shows the flowchart of the participants in this study as well as the biological samples and questionnaire information collected. *Sample size collected and response rate expressed as *n* (%).

A field survey was conducted in May 2021 in Xinjiang province. Counties in northern and southern Xinjiang were ranked separately according to their economic levels, and one county with a high level of economic development and one county with a relatively low level of economic development were selected as field survey sites in each of the northern and southern regions using the random number table method. Specifically, Yining (high level of economic development) and Toli (low level of economic development) counties were chosen in northern Xinjiang, and Korla (high level of economic development) and Wushi (low level of economic development) counties were chosen in southern Xinjiang. Each site was divided into five sampling areas in different geographic locations (east, west, south, north and center), and twenty households were randomly selected from the commune/street in each of the five areas, giving a total of 100 households sampled at each site. Biological samples were collected from one adult in each household, with equal numbers of samples taken from males and females (Fig. 1). Inclusion criteria: (1) local residents who had lived in the area for at least 5 years and were aged at least 18 years; (2) no history of thyroid disease, autoimmune disease, endocrine disease or familial genetic disease. Exclusion criteria: (1) pregnant and lactating women; (2) people with occupational exposure to iodine (e.g. medical personnel using iodine disinfectant and iodine contrast medium) or (3) people who had recently used iodine-containing lotion, taken Wassail tablets, or had recently undergone a contrast examination.

### Questionnaire design and evaluation

Given the dietary habits of Xinjiang residents and the iodine content of foods marketed in Xinjiang, we used a customised FFQ suitable for evaluating iodine nutrition levels in Xinjiang adults, taking into account the iodine contribution of various foods^(12)^. The questionnaire was divided into two parts: the first part collected the basic information of the respondents, including age, sex, body weight, history of hypertension, history of thyroid disease and salt consumption (iodised salt or non-iodised salt), while the second part was a survey on the frequency and amount of food intake, which was further divided into thirteen main categories, namely staple foods, soya products, meat, eggs, milk, aquatic products, mushrooms and algae, vegetables, offal, snacks, nuts, condiments and iodised salt intake. The grouping of foods is shown in Table 1. Food frequency was set to five levels: ≥ 1 time/d, 1–6 times/week, 1–3 times/month, 1–5 times/half year and never. The amount of intake each time was recorded in grams. The average daily food intake (g/d) was calculated as (number of times of eating × amount of intake each time) ÷ number of days included in the cycle. The FFQ were conducted face-to-face by uniformly trained enumerators with reference to quantitative standards of food intake stipulated in the Dietary Review Survey Supplementary Reference Food Chart^(13)^.

**Table 1.** Food groups used in the dietary analysis according to their nutritional composition and taxonomy

No.	Food group	Food items
1	Noodles	Noodles, hanging noodles, Ramen
2	Rice	Rice, thin rice, brown rice, aromatic rice
3	Beans and products	Soya products, flavoured soy products
4	Meats	Livestock and poultry meat, processed meat products
5	Eggs	Eggs, duck eggs, quail eggs, goose eggs, brined eggs
6	Dairy	Erie Schmaltz milk, flavoured yoghurt, fresh milk
7	Fish, shrimp and shellfish	Marine products
8	Offtake of fungi and algae	Seaweed, seaweed, nori, fungus, seaweed flavoured snacks
9	Vegetables	Lotus root, white radish, carrot, chilli, aubergine, etc.
10	Fruits	Apples, bananas, pears, etc.
11	Nuts	Walnuts, pistachios, Pine nuts, almonds
12	Condiments	Soy sauce, balsamic vinegar, ground ginger, ketchup, shrimp paste
13	Salt	Iodized salt

### Sample collection and laboratory testing

A plastic tube with a screw cap was used to collect a one-time random urine sample of 5 ml from each subject, which was then transported to the laboratory under ambient conditions and stored in a refrigerator at 4–8°C. The urinary iodine concentration (UIC) was measured using the WST107.1-2016 protocol ‘Determination of iodine in urine iodine, Part 1: Arsenic–cerium catalytic spectrophotometric method’. Subsequently, 5 ml of venous blood sample from each subject was collected in a 5 ml clear plastic freezing tube with a screw cap by the investigator according to the requirements for aseptic blood collection and left to stand for 30 min at room temperature. Subsequently, the blood samples were centrifuged at 3000 rpm (radius of 15 cm) for 15–20 min, and the supernatant (serum) of ≥ 2 ml was collected. Serum concentrations of free triiodothyronine (FT3), free thyroxine (FT4), and thyrotropin were measured using a Roche E411 electrochemiluminescence immunoassay analyser (Roche, cabas e411).

### Criteria for determination

According to the median urinary iodine standards for adults recommended by the WHO, the UNICEF and the International Council for the Control of Iodine Deficiency Disorders (Iodine Global Network) in 2007, a median UIC < 100 μg/l is considered iodine deficient, 100–199 μg/l is considered iodine appropriate (optimal), 200–299 μg/l is considered iodine super-appropriate and ≥ 300 μg/l is considered iodine excess. The Roche Electrochemical Immunoassay Thyroid Function Reference Values were referred to for the normal serum concentration ranges of thyroid hormones in adults: FT3: 3.1–6.8 pmol/l, FT4: 12–22 pmol/l and thyrotropin: 0·27–4·2 µIU/l.

### Data analysis

IBM SPSS Statistics 26.0 and SAS 9.4 software were used to statistically analyse the data. Data were tested for normality by the Kolmogorov–Smirnov method, and normally distributed data were statistically expressed as 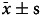 . An ANOVA was used to compare normally distributed variables between groups. For variables with a skewed distribution, median and interquartile spacing were used for statistical description with median (interquartile range), and between-group comparisons were performed using the Wilcoxon rank sum test. Principal component analysis (PCA) was used for factor analysis to obtain a more realistic factor structure, ensuring that the distribution of the factor scores was centered on 0 with a sd of 1. All food groups in the FFQ were included in the analysis. Variance-maximising orthogonal rotations were used to calculate the factor loadings, which represent the standardised correlation between the factor and the food group. Subsequently, dietary pattern scores were calculated. If the score is <0, it indicates that the individual is below the average level compared with all other participants; if the pattern score equals 0, it indicates that the individual has a level consistent with the average; if the pattern score is greater than 0, it indicates that the individual is above the average level. The factor loadings were then combined with the eigenroots, the variance contribution ratio and the reasonableness of food modeling to finalise the dietary model. The factor scores from the PCA were then used to determine the factor scores of each sample in each dietary model, and the correlation analyses of the dietary model scores with UIC and thyroid function were performed using Spearman’s correlation. Finally, quartiles (Q1 (lowest intake), Q2, Q3 and Q4 (highest intake)) were constructed for each factor score, and binary logistic regression was used to analyse the relationship between dietary patterns and iodine nutrition levels. In the regression model, the ‘dietary pattern’ score is treated as the independent variable, while the ‘age and BMI’ of adults are used as control variables, and ‘iodine deficiency status’ serves as the dependent variable. The significance level for statistical analysis was set at 0·05.

## Results

### Participant characteristics

A total of 435 adults were included in this survey, of whom 46·44 % were female and 53·56 % were male, with a mean age of 46·00 years. Most of the adults had a normal BMI (45·05 %). In addition, 82·30 % of the participants had no history of hypertension and 97·24 % had no history of thyroid disease. All participants consumed iodised salt (Table 2).

**Table 2.** Baseline characteristics of participants*

Characteristics	Units	Adults (*n* 435)	
Age(years)	Median (IQR)	46·00	34·00, 58·00
Sex			
Man	*n* (%)	202	46·44
Women	*n* (%)	233	53·56
BMI (kg/m^2^)^a^			
Underweight	*n* (%)	10	2·30
Normal	*n* (%)	196	45·06
Overweight	*n* (%)	131	30·11
Obesity	*n* (%)	98	22·53
With or without a job			
Yes	*n* (%)	365	83·91
No	*n* (%)	70	16·09
History of hypertension			
Yes	*n* (%)	77	17·70
No	*n* (%)	358	82·30
History of thyroid disease			
Yes	*n* (%)	12	2·76
No	*n* (%)	423	97·24
Iodised salt consumption			
Iodised salt	*n* (%)	435	100·00
Non-iodised salt	*n* (%)	0	0·00

IQR, interquartile range.

*Data are expressed as median (IQR), or *n* (%); ^a^BMI: is calculated from adult weight and height. BMI < 18·5 kg/m^2^: thin; 18·5 ≤ BMI < 24·0 kg/m^2^: normal; 24·0 ≤ BMI < 28·0 kg/m^2^: overweight; BMI ≥ 28·0 kg/m^2^: obese; housewives were defined as unemployed.

### Characteristics of dietary patterns

The Kaiser–Meyer–Olkin measure value of factor suitability for PCA was 0·597, which is >0·5, indicating that the food groups set in this study were suitable for PCA. Bartlett’s spherical test showed a *χ* ^*2*^ of 689·112, with *P* < 0·001, indicating that the food groups were not independent of each other and had strong correlations that allowed for factor analysis. Eigenvalues greater than the 1·0 criterion were selected, and the number of factors was determined to be four based on the fragmentation plot and interpretable variance. The selected components were named according to factor loadings > 0·5, and the retained dietary patterns were labelled as follows: PCA1: the staple food pattern (predominantly pasta, rice and salt), PCA2: the vegetable, fruit, and meat pattern (predominantly vegetables, nuts, fruits and meat), PCA3: the fish, shrimp and legume pattern (predominantly fish, shrimp and shellfish, legumes and mushrooms) and PCA4:the dairy-based protein pattern (predominantly eggs and milk). The cumulative contribution of the four dietary patterns was 51·874 %, with the highest variance contribution of 14·59 % demonstrated by the staple food pattern, followed by 13·90 % by the fruit, vegetable and meat pattern; 13·35 % by the fish, shrimp and legume pattern and 10·03 % by the dairy-based protein pattern (Table 3).

**Table 3.** Determination of factor loadings for food groups in each dietary pattern using PCA^*^

Foods	PCA1	PCA2	PCA3	PCA4
Noodles	0·79	0·15	−0·05	0·12
Rice	0·72	−0·05	0·28	−0·17
Salt	0·61	−0·22	0·07	0·25
Vegetables	−0·16	0·68	0·23	−0·10
Nuts	−0·10	0·69	−0·10	0·26
Fruits	0·17	0·59	0·35	−0·15
Meats	0·45	0·55	−0·10	−0·07
Fish, shrimp and shellfish	0·01	−0·02	0·70	0·29
Beans and products	0·19	−0·16	0·65	0·11
Offtake of fungi and algae	−0·01	0·23	0·56	−0·16
Eggs	−0·04	−0·13	0·25	0·82
Diary	0·31	0·19	−0·21	0·55
Condiments	0·01	0·12	0·35	−0·04

PCA, principal component analysis.

* Extraction method: principal component analysis; rotation method: orthogonal rotation with Kaiser standardisation.

### Correlation of dietary patterns with urinary iodine concentrations and thyroid function

Spearman’s correlation analyses were performed to assess the relationship of dietary pattern scores with UIC and thyroid function in adults. The results showed that scores of PCA1 were positively correlated with the UIC (*r* = 0·41, *P* < 0·05); scores of PCA2 were negatively correlated with both the FT4 concentration and UIC (*r* = –0·14 and –0·45, respectively, both *P* < 0·05); scores of PCA3 were positively correlated with the UIC (*r* = 0·30, *P* < 0·05) and scores of PCA4 were negatively correlated with the FT4 concentration (*r* = –0·11, *P* < 0·05). The results are displayed in Table 4.

**Table 4.** Correlation of PCA factor scores with UIC and thyroid function

Dietary patterns		FT3(*n* 419)	FT4(*n* 412)	TSH(*n* 390)	UIC(*n* 435)
PCA1	*r*	0·09	−0·04	−0·03	0·41
	*P*	0·079	0·060	0·595	< 0·001
PCA2	*r*	−0·09	−0·14	0·07	−0·45
	*P*	0·067	0·004	0·202	< 0·001
PCA3	*r*	0·01	−0·01	−0·01	0·30
	*P*	0·900	0·923	0·782	< 0·001
PCA4	*r*	0·11	−0·11	−0·01	0·00
	*P*	0·059	0·030	0·887	0·966

PCA, principal component analysis; UIC, urinary iodine concentration.

### Relationship between different urinary iodine concentrations and thyroid function

The differences in serum thyrotropin concentrations between the four UIC groups were not statistically significant (*P* > 0·05), while those in serum FT3 and FT4 concentrations were statistically significant (*P* < 0·05). The serum FT3 and FT4 concentrations in the iodine-excess group were significantly higher than those in the iodine-appropriate group (*P* < 0·05; Table 5).

**Table 5.** Thyroid hormone levels in adults with different urinary iodine concentrations*

Thyroid hormone	Urine iodine concentration(μg/l)								*H*/*F*	*P*
	< 100 (*n* 34)		100–199 (*n* 140)		200–299 (*n* 157)		≥ 300 (*n* 104)			
	Mean	sd	Mean	sd	Mean	sd	Mean	sd		
TSH/(mIU/l)	2·24	1·46, 3·30	2·09	1·37, 3·37	2·21	1·20, 3·05	1·89	1·38, 2·97	1·457	0·692
	Median	IQR	Median	IQR	Median	IQR	Median	IQR		
FT3/(pmol/l)	5·30	0·16	5·24	0·06	5·30	0·07	5·53	0·07†	2·861	0·037
FT4/(pmol/l)	15·80	0·52	15·87	0·21	16·19	0·21	16·64	0·22†	2·667	0·047

TSH, thyrotropin; IQR, interquartile range; FT3, triiodothyronine; FT4, free thyroxine.

*TSH is expressed as M (IQR) and FT3 and FT4 as mean (sd); †*P* < 0·05 (compared with iodine-appropriate group).

### Multivariable analysis of dietary patterns and urinary iodine concentrations in adults

To further explore the relationship between dietary patterns and UIC, logistic regression analyses were performed with dietary patterns as the independent variable and sex, age and BMI as the control variables. The results showed that in PCA1, compared with the group with the highest dependence (Q4), the remaining quartiles (Q1, Q2 and Q3) were associated with an increased risk of iodine deficiency (UIC < 100 μg/l) (OR = 260·41, 59·86, and 2·01, respectively, all *P* < 0·05); in PCA2, compared with Q4, Q3 was associated with an increased risk of iodine deficiency (OR = 0·16, *P* < 0·05); in PCA3, compared with Q4, Q3 was associated with an increased risk of iodine deficiency (OR = 0·23, *P* < 0·05) and in PCA4, the lowest dependence (Q1) was associated with an increased risk of iodine deficiency compared with Q4 (OR = 31·30, *P* < 0·05), and no correlation was seen among the remaining quartiles. The results are displayed in Table 6.

**Table 6.** Risk ratios (OR) and 95 % confidence intervals for iodine deficiency and dietary patterns (DPS) in adults*

Dietary patterns	Q1 (*n* 38)		Q2 (*n* 97)		Q3 (*n* 145)		Q4 (reference) (*n* 155)	*P*
	OR	95 % CI	OR	95 % CI	OR	95 % CI		
PCA1	260·41†	20·16, 663·70	59·89†	5·64, 335·81	2·01†	0·15, 26·16	1	< 0·001
PCA2	0·01	0·00, 0·10	0·02	0·00, 0·12	0·16†	0·05, 0·53	1	< 0·001
PCA3	2·79	0·55, 14·27	0·46	0·10, 2·10	0·23†	0·06, 0·90	1	0·034
PCA4	31·30†	4·88, 200·64	5·84	0·97, 35·17	0·79	0·10, 6·02	1	< 0·001

*Data are expressed as OR and 95 % confidence intervals for OR; †*P* < 0·05.

## Discussion

The aim of this study was to understand the dietary patterns of the adult population and to explore their associations with iodine nutrition levels and thyroid function. First, we identified four dietary patterns in adults in Xinjiang through PCA. Subsequently, assessment of the association of dietary patterns with UIC and thyroid function revealed that PCA1 (the staple food pattern (predominantly pasta, rice and salt)) and PCA3 (the fish, shrimp and legume pattern (predominantly fish, shrimp, and shellfish, legumes and mushrooms)) were positively correlated with UIC; PCA2 (the vegetable, fruit, and meat pattern (predominantly vegetables, nuts, fruits, and meat)) was negatively correlated with UIC, and the association of dietary patterns with thyroid function was NS. Finally, the relationship between dietary patterns and UIC was explored, and it was found that low dependence (Q1) was associated with an increased risk of iodine deficiency compared with the highest dependence (Q4) for each dietary pattern.

Between 1997 and 2009, Chinese adults had four dietary patterns: the northern pattern (based on flour, cereals and potatoes), the southern pattern (based on rice, vegetables and meat), the fast-food pattern (based on fast food, dairy and snacks) and the snacking pattern (based on dairy, fruits and eggs)^(14)^. However, in recent years, with the development of society, the dietary structure of the population has also changed. A study conducted in 2021 showed that the dietary structure of the Chinese population is becoming increasingly diversified; although the current traditional pattern (mainly rice, coarse and mixed grains, vegetables, livestock and meat and fruit intake) accounts for a high proportion, increasing numbers of people have been adopting Mediterranean dietary patterns or balanced dietary patterns^(15,16)^. Xinjiang is in northwestern China (encompassing Qinghai, Ningxia, Shanxi, Gansu and Xinjiang), where studies have shown that the dietary structure of the population is generally imbalanced with pasta, fruits and vegetables as the main food groups and a high intake of salt and meat products, while the intake of dietary fibre, vitamins and dairy products is lower than their recommended levels^(16)^. This finding in line with the results of this study, indicating that differences in dietary patterns are closely related to geography, climate, economy, dietary habits and cultural background. Seafood and seaweeds are a good source of iodine, but Xinjiang is geographically far from the ocean. Accordingly, such foods were not present in any of the main dietary patterns of the Xinjiang population in this study. Thus, staple foods, fruits and vegetables, livestock and poultry meats and dairy products can be regarded as the main food sources of iodine for the population.

Dietary pattern scores are continuous variables and therefore can be used for subsequent correlation analyses. We used Spearman’s correlation analyses to preliminarily explore the relationship between dietary patterns and thyroid function and UIC in adults, which showed that PCA1 and PCA3 were positively correlated with the UIC, whereas PCA2 was weakly negatively correlated with the UIC. Healthy dietary choices are essential for adequate iodine intake. In countries where salt iodisation is not mandatory, such as the United Kingdom and Japan, the main dietary sources of iodine are marine fish, seafood, seaweeds and dairy products, whereas in most countries, the main dietary source of iodine is iodised salt^(17)^. In the present study, the staple food pattern contained nuts, pasta, livestock and poultry meat, rice and iodised salt, and the fish, shrimp and legume pattern contained fish, shrimp and shellfish, legumes and mushrooms. Our results showed that both of these dietary patterns were positively correlated with the UIC, which is likely to be related to the types of food included in these dietary patterns. In this study, seaweed foods such as kelp, nori and seaweed were categorised as mushrooms, and the pattern comprising fish, seafood and mushrooms showed a correlation with the UIC, which is consistent with the results of another study^(18)^. In addition, iodised salt, poultry eggs and dairy are good sources of dietary iodine in the protein and staple food patterns. A dietary survey study showed that the UIC increased with increased intake of dairy products and eggs, and that dairy products were the best food source for improving the UIC^(19,20)^. Furthermore, no statistical correlation was seen between the dairy-based protein pattern and UIC in this study. In fact, milk is naturally low in iodine, and some studies have shown that most of the iodine in milk comes from animal feed and indirect fortification with iodine-containing disinfectants and that seasonality and farming practices and processing affect the iodine concentration of milk; some other studies have also suggested that prolonged use of nonconventional milk puts people at risk of iodine deficiency^(21,22)^. Residents of the Xinjiang region live in rural areas with abundant raw materials and tend to consume homemade dairy and meat products more frequently than seafood. In addition to the above dietary patterns, the dietary patterns of fruits and vegetables showed a weak negative correlation with the UIC in adults, which is consistent with the results of the National Health and Nutrition Examination Survey study from 2007 to 2012^(23)^. The reasons for the above results are considered to be related to the limited knowledge and awareness of iodine nutrition among the survey respondents, as well as the low preference for iodine-rich foods. A previous survey conducted in Xinjiang showed that the respondents’ knowledge of iodine nutrition was low, with vegetables, fruits and meats incorrectly considered as iodine-rich foods by some respondents and some not knowing what foods were good sources of iodine^(24)^. A survey in China showed that the UIC increased significantly with increasing iodine knowledge scores^(25)^. A study in Shanghai also showed that maternal iodine-related knowledge and behaviors were associated with iodine intake^(26)^. Although the iodine-related knowledge, attitudes and behaviours of the study participants were not investigated in the present study, our results combined with those of previous research^(24)^ suggest the need to educate the public on the importance of and ways to improve iodine nutrition for health.

Numerous studies have shown that iodine intake affects thyroid function^(27,28)^. In the present study, the UIC was found to be correlated with thyroid function, while no significant correlation was found between the dietary patterns and thyroid function; rather, the dietary patterns were found to mainly affect the UIC. Some epidemiological studies have found that dietary intake (independent of iodine) may also influence thyroid function Iacovides *et al.* found that a ketogenic diet decreased serum T3 concentrations and increased serum T4 concentrations, whereas a high-carbohydrate, low-fat diet did not affect thyroid function^(29)^. Basolo *et al.* reported that a high-protein diet decreased thyrotropin, FT3 and FT4 concentrations, whereas a low-protein diet decreased the plasma concentrations of thyroid-stimulating hormone and increased the plasma concentrations of FT3^(30)^. The dietary pattern comprising fruits, vegetables and meat was weakly negatively correlated with FT4 concentrations in adults in this study, consistent with the results of a survey of the Mediterranean diet and thyroid function^(31)^. This finding in our study may be related to the relatively small proportion of adults who preferred iodine-rich foods in the fruit and vegetable dietary pattern. This assumption needs to be further investigated in a larger sample size. Numerous studies have shown that thyroid function is affected by a wide range of factors, including genetic factors, dietary and lifestyle habits, physical and chemical factors, ionising radiation and environmental endocrine disruptors^(32–34)^. Daily diet can also be influenced by environmental factors, making it difficult to judge the direct relationship between dietary patterns and thyroid function.

The results of the relationship between dietary patterns and UIC indicated that Q1, Q2 and Q3 in the staple food pattern were all associated with an increased risk of iodine deficiency disorders compared with the highest dependence quartile (Q4), suggesting that the more adults tend to eat pasta, rice and iodised salt the less likely they are to develop iodine deficiency. For the fruit, vegetable and meat pattern and the fish, shrimp and soya pattern, only Q3 was associated with an increased risk of iodine deficiency disorders compared with the highest dependence quartile (Q4), and no association was found between the lowest dependence quartile (Q1) and iodine deficiency disorders. This may be related to the preponderance of major food groups in the above two dietary patterns; seafood such as fish, shrimp and shellfish and mushrooms are iodine-rich foods, and although soya products and cruciferous vegetables contain substances such as isoflavones and bisulfates that inhibit the absorption of thyroid hormones^(35)^, with a balanced diet such dietary patterns may not have a significant effect on the UIC. In contrast, an increased risk of iodine deficiency was found in the dairy-based protein pattern in the lowest dependence quartile (Q1) compared with the highest dependence quartile (Q4), suggesting that those who preferred the dairy-based protein pattern were less susceptible to iodine deficiency.

The above results suggest that the dietary pattern mainly affects the UIC, while its effect on thyroid function is NS. Although our results were derived using statistical methods, more studies are needed to validate our findings. The strength of this study is that it is the first to explore the association of dietary patterns with iodine nutrition levels and thyroid function. In addition, the participants were adults from two different geographical areas of Xinjiang province, making the sample representative of that region. In addition to dietary data, data on urinary iodine and thyroid function were also obtained. The study also has some limitations. First, the estimation of dietary intake by the FFQ is limited by recall bias and possible omission of food groups from the questionnaire^(36)^. However, the FFQ has been widely used in numerous studies to measure typical dietary exposures and behaviours and as a reasonably reproducible and valid tool for assessing overall dietary consumption using a dietary pattern approach^(37)^. Second, PCA, as a dimensionality reduction method involving subjective judgment, is typically limited by subjective bias on the part of the researcher in interpreting factor analysis^(38)^. Third, while the UIC is a good indicator to evaluate the iodine intake of the populations, a single random UIC cannot be used to represent the iodine nutritional status of individual due to the susceptibility of drinking water and foods^(39)^. Fourth, given the cross-sectional nature of this study, causality could not be inferred. Therefore, prospective studies with larger sample sizes are recommended in the future.

### Conclusion

In summary, the staple food pattern identified by the PCA method, characterised by a predominance of pasta, rice and iodised salt, was found to be positively associated with the UIC, and the lowest dependence (Q1) was associated with an increased risk of iodine deficiency compared with the highest adherence (Q4). Similarly, the fish, shrimp, shellfish and legume pattern, characterised by a predominance of fish, shrimp, shellfish, legumes and mushrooms, was also positively associated with the UIC and associated with an increased risk of iodine deficiency disorders in Q3 compared with the highest adherence quartile (Q4). In addition, the dairy-based protein pattern, characterised by a predominance of eggs and dairy, was not found to be correlated with the UIC, but in multivariable analyses, it was found to be associated with an increased risk of iodine deficiency disorders in the lowest dependence quartile (Q1) compared with Q4. However, the current evidence on the effect of dietary patterns on thyroid function needs to be supported by further longitudinal studies that include long-term follow-up, larger sample sizes and repeated measures.

## References

[1] Chen H & Calder PC (2020) Defining a healthy diet: evidence for the role of contemporary dietary patterns in health and disease. Nutrients 12, 334. 10.3390/nu12020334. 32012681 PMC7071223

[2] Kelly OJ , Gilman JC & Ilich JZ (2018) Utilizing dietary micronutrient ratios in nutritional research may be more informative than focusing on single nutrients. Nutrients 10, 107. 10.3390/nu10010107. 29351249 PMC5793335

[3] Zhao J , Li Z , Gao Q et al. (2021) A review of statistical methods for dietary pattern analysis (J). Nutr J 20, 37. 10.1186/s12937-021-00692-7. 33874970 PMC8056502

[4] Zimmermann MB (2009) Iodine deficiency. *Endocr Rev* **30**, 376–408. 10.1210/er.2009-0011. 19460960

[5] Pearce EN , Lazarus JH , Moreno-Reyes R et al. (2016) Consequences of iodine deficiency and excess in pregnant women: an overview of current knowns and unknowns. Am J Clin Nutr 104, 918S–923S. 10.3945/ajcn.115.110429. 27534632 PMC5004501

[6] Zimmermann MB , Jooste PL & Pandav CS (2008) Iodine-deficiency disorders. Lancet 372, 1251–1262. 10.1016/S0140-6736(08)61005-3. 18676011

[7] Melse-Boonstra A & Jaiswal N (2010) Iodine deficiency in pregnancy, infancy and childhood and its consequences for brain development. Best Pract Res Clin Endocrinol Metab 24, 29–38. 10.1016/j.beem.2009.09.002. 20172468

[8] Bath SC (2019) The effect of iodine deficiency during pregnancy on child development. Proc Nutr Soc 78, 150–160. 10.1017/S0029665118002835. 30642416

[9] Toloza FJ , Motahari H & Maraka S (2020) Consequences of severe iodine deficiency in pregnancy: evidence in humans. Front Endocrinol 11, 409. 10.3389/fendo.2020.00409. PMC7318882 32636808

[10] Liu T , Li Y , Teng D et al. (2021) The characteristics of iodine nutrition status in china after 20 years of universal salt iodization: an epidemiology study covering 31 provinces. Thyroid 31, 1858–1867. 10.1089/thy.2021.030. 34806437

[11] Guo Y , Zynat J , Xu Z et al. (2016) Iodine nutrition status and thyroid disorders: a cross-sectional study from the Xinjiang Autonomous Region of China. Eur J Clin Nutr 70, 1332–1336. 10.1038/ejcn.2016.82. 27188916

[12] Li S , Guo W , Ren Z et al. (2023) The simplified iodine-specific food frequency questionnaire can evaluate iodine intake in Chinese adults. Nutr Res 109, 47–57. 10.1016/j.nutres.2022.12.001. 36586289

[13] Wang Z , Zhang M , Wu J et al. (2014) Study on establishment and evaluation of a novel method for dietary assessment with instant photography. Acta Nutrimenta Sin 36, 288–295. 10.13325/j.cnki.acta.nutr.sin.2014.03.018.

[14] Zhang JG (2014) Changes in Dietary Patterns and their Association with General and Central Obesity among Adults in China (1991–2009); Chinese Centre for Disease Control and Prevention. (in Chinese).

[15] Du R & Cao H (2021) Trajectories of Mediterranean diet adherence and risk of metabolic syndrome in Chinese adults: results from the CHNS study, 1997–2009. Mod Prev Med 48, 2734–7+54. (in Chinese).

[16] Ma Z , Hao X , Wang D et al. (2023) Evolution and distribution of dietary patterns in China and the research progress of its correlation with health. Food Ind Sci Technol 44, 396–405. 10.13386/j.issn1002-0306.2022060202.

[17] Bouga M , Lean MEJ & Combet E (2018) Contemporary challenges to iodine status and nutrition: the role of foods, dietary recommendations, fortification and supplementation. Proc Nutr Soc 77, 302–313. 10.1017/s0029665118000137. 29704906

[18] Aakre I , Tveito Evensen L , Kjellevold M et al. (2020) Iodine status and thyroid function in a group of seaweed consumers in Norway. Nutrients 12, 3483. 10.3390/nu12113483. 33202773 PMC7697291

[19] Gostas DE , Larson-Meyer DE , Yoder HA et al. (2020) Dietary relationship with 24 h urinary iodine concentrations of young adults in the Mountain West Region of the United States. Nutrients 12, 121. 10.3390/nu12010121. 31906335 PMC7019367

[20] Adalsteinsdottir S , Tryggvadottir EA , Hrolfsdottir L et al. (2020) Insufficient iodine status in pregnant women as a consequence of dietary changes. Food Nutr Res 64. 10.29219/fnr.v64.3653. PMC6958617 31983913

[21] Ma W , He X & Braverman L (2016) Iodine content in milk alternatives. Thyroid 26, 1308–1310. 10.1089/thy.2016.0239. 27358189

[22] Van Der Reijden OL , Galetti V , Bürki S et al. (2019) Iodine bioavailability from cow milk: a randomized, crossover balance study in healthy iodine-replete adults. Am J Clin Nutr 110, 102–110. 10.1093/ajcn/nqz092. 31788697

[23] Torres MT , Vila L , Manresa JM et al. (2020) Impact of dietary habit, iodine supplementation and smoking habit on urinary iodine concentration during pregnancy in a Catalonia population. Nutrients 12, 2656. 10.3390/nu12092656. 32878172 PMC7551663

[24] Nie J , Zhu Y , Wang C et al. (2023) Relationship between iodine knowledge and dietary iodine intake in pregnant and lactating women: a cross-sectional study. Public Health Nutr 26, 1436–1450. 10.1017/s1368980023000514. 36946300 PMC10346033

[25] Wang X , Lou X , Mo Z et al. (2019) Poor iodine knowledge, coastal region, and non-iodized salt consumption linked to low urinary iodine excretion in Zhejiang pregnant women. Nutrients 11, 413. 10.3390/nu11020413. 30781393 PMC6412776

[26] Tian W , Yan W , Liu Y et al. (2021) The status and knowledge of iodine among pregnant women in Shanghai. Biol Trace Elem Res 199, 4489–4497. 10.1007/s12011-021-02587-4. 33462796

[27] Wu Y , Yang J , Su Q et al. (2023) Urinary iodine concentration and its associations with thyroid function in pregnant women of Shanghai. Front Endocrinol (Lausanne) 14, 1184747. 10.3389/fendo.2023.1184747. 37469986 PMC10352823

[28] Sun R , Fan L , Du Y et al. (2022) The relationship between different iodine sources and nutrition in pregnant women and adults. Front Endocrinol (Lausanne) 13, 924990. 10.3389/fendo.2022.924990. 35983514 PMC9379486

[29] Iacovides S , Maloney SK , Bhana S et al. (2022) Could the ketogenic diet induce a shift in thyroid function and support a metabolic advantage in healthy participants? A pilot randomized-controlled-crossover trial. PLoS One 17, e0269440. 10.1371/journal.pone.0269440. 35658056 PMC9165850

[30] Basolo A , Begaye B , Hollstein T et al. (2019) Effects of short-term fasting and different overfeeding diets on thyroid hormones in healthy humans. Thyroid 29, 1209–1219. 10.1089/thy.2019.0237. 31298652 PMC6864752

[31] Zupo R , Castellana F , Panza F et al. (2020) Adherence to a Mediterranean Diet and thyroid function in obesity: a cross-sectional Apulian survey. Nutrients 12, 3173. 10.3390/nu12103173. 33081337 PMC7603040

[32] Ruggeri RM , Giovinazzo S , Barbalace MC et al. (2021) Influence of dietary habits on oxidative stress markers in Hashimoto’s thyroiditis. Thyroid 31, 96–105. 10.1089/thy.2020.0299. 32729374

[33] Gruppen EG Kootstra-Ros J , Kobold AM et al. (2020) Cigarette smoking is associated with higher thyroid hormone and lower TSH levels: the PREVEND study. Endocrine 67, 613–622. 10.1007/s12020-019-02125-2. 31707605 PMC7054375

[34] Bellastella G , Scappaticcio L , Caiazzo F et al. (2022) Mediterranean diet and thyroid: an interesting alliance. Nutrients 14, 4130. 10.3390/nu14194130. 36235782 PMC9571437

[35] Messina M & Redmond G (2006) Effects of soy protein and soybean isoflavones on thyroid function in healthy adults and hypothyroid patients: a review of the relevant literature (J). Thyroid 16, 249–258. 10.1089/thy.2006.16.249. 16571087

[36] Shakya PR , Melaku YA , Page A et al. (2020) Association between dietary patterns and adult depression symptoms based on principal component analysis, reduced-rank regression and partial least-squares. Clin Nutr 39, 2811–2823. 10.1016/j.clnu.2019.12.011. 32007317

[37] Liu X , Wang X , Lin S et al. (2015) Reproducibility and validity of a food frequency questionnaire for assessing dietary consumption via the dietary pattern method in a Chinese Rural Population. PLoS One 10, e0134627. 10.1371/journal.pone.0134627. 26230275 PMC4521698

[38] Zhang F , Tapera TM & Gou J (2018) Application of a new dietary pattern analysis method in nutritional epidemiology. BMC Med Res Methodol 18, 119. 10.1186/s12874-018-0585-8. 30373530 PMC6206725

[39] Wainwright P & Cook P (2019) The assessment of iodine status – populations, individuals and limitations. Ann Clin Biochem 56, 7–14. 10.1177/0004563218774816. 29703103

